# AI-Assisted Handheld Echocardiography by Nonexpert Operators: A Narrative Review of Prospective Studies

**DOI:** 10.7759/cureus.97050

**Published:** 2025-11-17

**Authors:** Hakeem A Shittu, Precious S Quaye

**Affiliations:** 1 Medicine and Surgery, Bedford Hospital, Bedford, GBR; 2 Medicine and Surgery, University Hospital Coventry & Warwickshire, Coventry, GBR

**Keywords:** artificial intelligence, education, ejection fraction, guidance, handheld echocardiography, image acquisition, novices, point-of-care ultrasound

## Abstract

Handheld point-of-care ultrasound (POCUS) increasingly incorporates AI to assist nonexpert operators in echocardiography, guiding image acquisition and automating core measurements. This narrative review synthesizes recent (2018-2025) prospective studies evaluating AI-assisted handheld echocardiography performed by nonexpert users in adult, pediatric, ambulatory, and critical care settings. A structured PubMed/MEDLINE search was used to identify eligible studies that included prospective clinical trials and randomized controlled trials that assessed real-time AI guidance, image quality feedback, automated view classification, and/or automated measurements (e.g., ejection fraction (EF) analysis). Across nine studies, AI guidance consistently improved image acquisition, enabling nonexperts to obtain diagnostically adequate transthoracic views in most patients after limited training. In hospital and outpatient settings, diagnostic adequacy for key triage questions such as left ventricular function and pericardial effusion frequently exceeded 90%. Automated EF analysis achieved close agreement with reference echocardiography, while end-to-end nurse-led pathways demonstrated feasible integration into routine clinic workflows with short examination times. Educational trials further showed higher view-acquisition success, faster learning, and improved recognition of systolic dysfunction with minimal time penalties. Overall, AI-assisted handheld echocardiography supports reliable, triage-oriented cardiac imaging by nonexperts across diverse environments. Strengths include enhanced feasibility, consistency, and efficiency, while Doppler-dependent tasks show lower adequacy and should not be frontline targets currently. Broader validation, outcome-based evaluation, and structured governance are needed to ensure safe, equitable, and scalable implementation.

## Introduction and background

The global burden of cardiovascular disease and echocardiography

Cardiovascular disease remains the leading cause of death worldwide. In 2021, it accounted for approximately 20.5 million deaths, around one-third of all global deaths, with more than three-quarters of these deaths occurring in low- and middle-income countries [1,2]. Forward projections indicate a substantial rise over the coming decades, with global cardiovascular deaths expected to exceed 35 million annually by 2050 if current trends continue [3].

Echocardiography is the cornerstone of noninvasive cardiac imaging. Compared with CT or MRI, it is portable, radiation-free, and relatively affordable, yet provides rich hemodynamic and structural information, such as ventricular size and function, chamber dimensions, valvular pathology, pericardial disease, and estimates of filling pressures [4]. Its clinical utility spans triage (e.g., undifferentiated dyspnea, chest pain, and shock) and longitudinal management (e.g., heart failure and valvular disease). However, echocardiography is highly operator-dependent, since acquiring diagnostic-quality images and interpreting them reliably requires extensive training and experience. In many systems, especially resource-constrained ones, the supply of skilled sonographers and cardiologists cannot meet demand, leading to delayed diagnosis, potentially avoidable morbidity, and persistent inequities in care access [5,6].

Rise of point-of-care ultrasound (POCUS) and handheld echocardiography

POCUS has transformed bedside assessment by bringing imaging to the patient and is now supported by consensus definitions and practice standards [7]. Handheld and portable ultrasound devices, some only the size of a smartphone, allow clinicians outside echo labs to perform focused cardiac assessments at the bedside, in ambulances, and in primary care. This shift allows for faster decisions and earlier triage, particularly where formal echocardiography is unavailable or delayed.

Yet POCUS echocardiography remains challenging for nonexpert operators, defined here as clinicians without formal echocardiography certification or specialist training. Standard transthoracic views needed to assess chamber size and function, such as the parasternal long-axis (PLAX), apical four-chamber (A4C), and subcostal views, require precise probe manipulation and real-time judgement of image quality. Subtle findings, such as regional wall-motion abnormalities, diastolic function, and valvular lesions, demand expertise. Short courses help but cannot eliminate the steep learning curve; therefore, variability in image quality and diagnostic accuracy persists among inexperienced operators [6]. These limitations have prompted interest in integrating AI to guide image acquisition and interpretation in POCUS, potentially expanding access to quality cardiac imaging.

AI in echocardiography

AI, especially machine learning and deep learning, has rapidly expanded echocardiography’s capabilities. Early clinical applications automated specific measurements such as left ventricular (LV) ejection fraction (EF), but modern tools add real-time view classification, image-quality feedback, and automated chamber quantification on-device. Recent clinical reviews detail how these capabilities are being deployed across acquisition, measurement, and reporting pipelines, and outline the translational barriers around validation and governance [8-10]. Many handheld platforms now embed AI modules that guide probe movement with on-screen cues (tilt/rock/rotate) to help nonexperts converge on standard views, score image quality in real time, prompt re-acquisition, label views automatically (e.g., A4C or PLAX), and quantify measurements such as EF or left atrial size while also screening for effusion [11].

**Figure 1:**
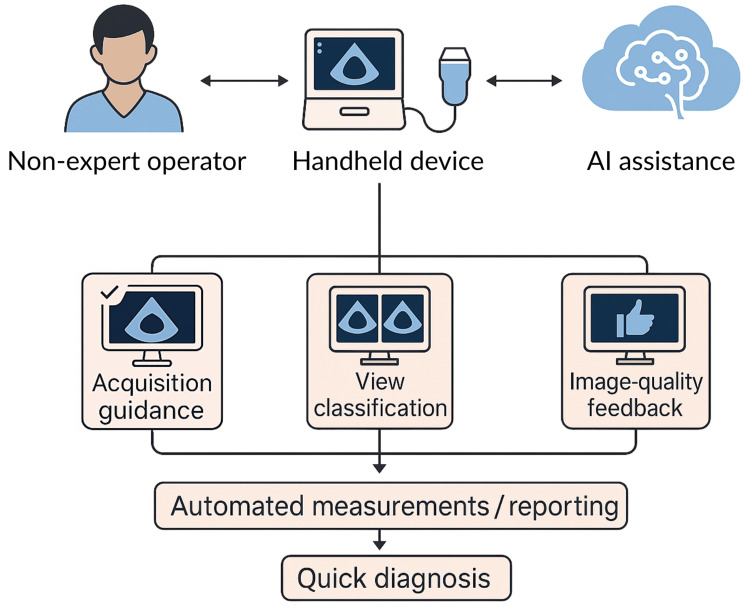
Conceptual workflow of AI-assisted handheld echocardiography A schematic representation of how AI integrates with handheld echocardiography. Nonexpert operators use handheld devices supported by AI assistance for image acquisition, view classification, and image-quality feedback. These processes feed into automated measurements and reporting (e.g., EF and chamber dimensions), leading to quicker diagnostic decision-making at the point of care. EF, ejection fraction Image created by authors using ChatGPT (Version 5.0; OpenAI, Inc., San Francisco, CA, USA)

Conceptually, such AI functions could raise the floor for nonexpert performance, making a larger proportion of studies diagnostically usable, and tightening variability across operators by standardizing the acquisition technique. This conceptual workflow is illustrated in Figure 1. The promise is not to replace expert echocardiography but to enable safe, focused, triage-oriented cardiac ultrasound by nonexperts while reserving full studies for complex or nonurgent cases [10].

The case for nonexpert-focused AI POCUS

The greatest potential impact of AI in echocardiography lies in empowering nonexpert users, who often encounter patients first and frequently practice where access to formal echocardiography is limited. In emergency and acute care, nurses or resident doctors can use AI-guided POCUS to confirm a large pericardial effusion/tamponade or severely reduced LV function within minutes. In critical care, ICU clinicians with limited echo training can assess RV dilation or LV systolic dysfunction at the bedside. In global health and pediatrics, community health workers in rheumatic heart disease (RHD)-endemic regions can screen for mitral disease with handheld devices. In primary care and outpatient triage, generalists can identify likely heart failure before referral. Collectively, these scenarios define the “last-mile” problem in cardiac imaging: bringing core echo capabilities to the point of need, safely and reproducibly, with nonexpert operators.

Evidence gap and rationale for review

Historically, much of the AI-in-echo literature centered on algorithm development or expert-acquired datasets, not real-world nonexpert use. In the last five years, however, prospective clinical studies and randomized trials have evaluated AI-assisted handheld echocardiography performed by nonexperts across adult and pediatric populations, reporting outcomes such as diagnostic adequacy, accuracy versus expert/reference echo, feasibility (view-acquisition success), efficiency (time), and workflow impact. A focused synthesis that isolates nonexpert operator evidence is therefore timely and clinically relevant.

Accordingly, this review summarizes prospective studies from 2018 to 2025 of AI-enabled handheld or portable echocardiography performed by nonexpert operators, compares diagnostic adequacy, accuracy, and workflow outcomes against expert benchmarks, highlights applications across adult, pediatric, and global settings, and discusses limitations, equity considerations, and research priorities needed for safe, scalable adoption.

## Review

Methods

Search Strategy

A structured literature search was conducted in PubMed/MEDLINE using combinations of controlled vocabulary and free-text terms related to echocardiography, handheld or portable ultrasound, AI, and nonexpert operators. An example Boolean query was (echocardiography OR “point-of-care” OR POCUS OR ultrasound) AND (handheld OR portable OR pocket) AND (“artificial intelligence” OR “machine learning” OR “deep learning” OR AI) AND (novice OR “nonexpert” OR nurse OR resident OR trainee OR student) AND (cardiac OR heart OR transthoracic OR TTE). Equivalent term variants were tested iteratively, and citation chaining (forward and backward reference searches) was used to identify additional relevant studies not fully indexed in PubMed at the time of search. We limited results to the English language, humans, and the date range January 1, 2018 to September 30, 2025, with the last search conducted on October 1, 2025. Given the heterogeneity of study designs, operator populations, and outcome measures, a narrative synthesis approach was chosen as the most appropriate method for summarizing and comparing the available evidence. No quantitative pooling or meta-analysis was performed.

Eligibility Criteria

Eligibility was defined prior to screening. These criteria were pre-specified to isolate prospective, real-world studies in which nonexpert operators used AI-enabled handheld or portable echocardiography. This approach was chosen to ensure methodological comparability across included studies and to exclude algorithm-development or expert-only work that falls outside the review’s clinical scope. We included studies in adults or children undergoing echocardiography performed by nonexpert operators (e.g., nurses, residents, students, paramedics, or community health workers) in which the intervention was AI-enabled handheld or portable echocardiography offering real-time acquisition guidance, view classification, image-quality assessment, or automated measurements such as EF. The comparator was an expert sonographer’s or cardiologist’s interpretation or a comprehensive reference echocardiogram. Outcomes of interest comprised diagnostic adequacy and accuracy, feasibility (success in acquiring standard views), efficiency (time), and workflow impact. Only prospective clinical designs, including randomized controlled trials, were eligible. We excluded noncardiac POCUS; studies limited to expert operators, algorithm development, simulation, or retrospective work without prospective nonexpert deployment; and non-peer-reviewed items such as preprints or conference abstracts.

Records were screened against the predefined criteria, and uncertainties were resolved by discussion. We performed a qualitative synthesis and tabulated key characteristics of the nine included prospective nonexpert operator studies.

Results

Adults and Outpatients

Real-time, on-device guidance is the most mature and clinically validated AI application for nonexpert echocardiography in outpatient and multicenter contexts. These systems continuously recognize probe position and current view and display actionable cues to help operators converge on standard transthoracic windows with adequate quality. In the pivotal trial by Narang et al., eight cardiac nurses with no prior ultrasound experience, following brief training, acquired 10 transthoracic views on 240 adults using an AI-guided handheld platform [12]. Blinded cardiologists judged the acquisitions to be adequate for diagnostic purposes in 92-98% of patients for core questions (LV size and systolic function, right ventricular size, and pericardial effusion), approximating expert-acquired references. In a multicenter trial, Mor-Avi et al. studied nurses and residents using real-time probe guidance across 240 adult studies and reported diagnostic sufficiency of 99.2% for LV size, 99.6% for LV function, 92.9% for RV size, and 100% for pericardial effusion, with qualitative interpretations from nonexpert-acquired images agreeing with expert-acquired images in 83-96% of cases [13]. An international study by Trost et al. further demonstrated that nonexpert nurses could complete a ten-view limited protocol on 240 adults with AI guidance, with all studies deemed sufficient for primary endpoints, including LV size, LV function, RV size, and presence of effusion. Automated quality gating and auto-capture features triggered recording only when predefined criteria were met, reducing the frequency of borderline clips that added little diagnostic value [14].

Beyond acquisition guidance, contemporary systems also automate interpretation tasks that have historically required substantial experience, most notably LV EF estimation and basic chamber quantification. In a large multicenter study (n = 449; 424 analyzed), Motazedian et al. reported an intraclass correlation coefficient of 0.904 for AI-derived EF versus comprehensive echocardiography (nonexperts: 0.921; experts: 0.845). Discriminative performance was excellent, with an area under the curve (AUC) of 0.98 for EF < 50% (sensitivity: 92.8%, specificity: 92.3%) and an AUC of 0.99 for EF < 30% (sensitivity: 78.1%, specificity: 98.0%) [15]. Performance was comparable between nonexperts and experts once analyzable views were available, indicating that automated quantification can equalize operator categories when acquisition is adequate. Huang et al. evaluated an end-to-end, nurse-led pathway incorporating AI guidance, automated EF analysis, and report generation in 100 clinic patients, where 96% of scans were interpretable and classification of EF < 50% achieved sensitivity of 84.6% and specificity of 91.4% (AUC 0.88). The mean absolute EF difference was 6% versus reference echocardiography, and the total examination time averaged 12.9 minutes, practical for ambulatory triage and surveillance [16].

Collectively, evidence in adult and outpatient settings shows that AI guidance substantially improves nonexpert acquisition, achieving diagnostic adequacy above 90% for key questions relating to LV function, RV size, and pericardial effusion. Automated EF quantification performs comparably to experts once the analyzable views are available. The main limitations arise under technically difficult acoustic windows, such as obesity, chronic obstructive pulmonary disease, or invasive ventilation, and for Doppler-based measurements that remain alignment-dependent.

ICU and Emergency Settings

In acute care, AI-assisted handheld echocardiography enables rapid bedside triage by nonexpert clinicians. Gallant et al. showed that ICU clinicians with limited ultrasound training obtained diagnostically viable PLAX and A4C views in approximately 96% of scans (80 scans across 10 ICU patients) within five minutes, with median acquisition times of 1.5 minutes for PLAX and 2.3 minutes for A4C [17]. Rapid acquisition of these high-yield views is clinically salient in shock or respiratory failure, wherein confirmation of large pericardial effusion, gross RV dilation, or markedly reduced LV function guides immediate management. In a separate accuracy cohort within the same study, AI-derived EF demonstrated high specificity (90%) but moderate sensitivity (56-70%) for identifying EF ≤ 40%, supporting reliable rule-in of marked systolic dysfunction while emphasizing the need for confirmatory assessment when the AI output appears normal despite ongoing clinical concern.

Overall, ICU and emergency data confirm that AI-guided handheld echocardiography allows nonexperts to obtain clinically actionable images within minutes, supporting early diagnosis and management in unstable patients. Limitations include modest sensitivity for moderate LV dysfunction and the persistent impact of suboptimal acoustic windows in mechanically ventilated or critically ill populations.

Education and Training Randomized Trials

Randomized educational studies support the role of AI as a practical teaching adjunct that accelerates learning and improves performance among nonexperts. In medical students performing handheld echocardiography, Karni et al. reported higher A4C success with real-time AI feedback (88% vs. 76%), with a modest 53-second increase in total examination time during early training [18]. In a single-center randomized trial of internal medicine residents (n = 43), Baum et al. observed that use of an AI-enabled handheld led to faster A4C acquisition (median 57 seconds vs. 85 seconds), higher blinded image-quality scores, and more accurate recognition of reduced LV systolic function (85% vs. 50%) compared with standard training [19]. In ambulatory pathways, the nurse-led approach evaluated by Huang et al. produced interpretable studies in 12.9 minutes on average with strong classification performance for EF below 50% [16].

Taken together, these studies demonstrate that AI feedback enhances the learning curve and diagnostic accuracy of trainees and nurses performing handheld echocardiography. Although early training may entail a small time penalty as users follow guidance meticulously, this is offset by fewer repeat scans, reduced need for expert rescue, and faster downstream decision-making. Sustained proficiency gains are contingent on structured onboarding and supervision frameworks.

Pediatric and Global Health Settings

AI-assisted nonexpert echocardiography is particularly relevant where trained sonographers are scarce. The Ugandan RHD screening study by Peck et al. illustrates this task-shifting potential, with diagnostic adequacy exceeding 90% for mitral valve morphology and regurgitation in community settings but lower adequacy for aortic valve assessments (approximately 79% for aortic regurgitation and 50% for aortic stenosis) due to Doppler alignment demands [20]. Although smaller thoracic dimensions and higher heart rates in children may facilitate image acquisition, outflow-tract and other Doppler-critical assessments remain challenging for nonexperts.

In summary, AI guidance enables nonexpert health workers to perform feasible pediatric and community-level echocardiographic screening, particularly for mitral pathology. Limitations include the technical difficulty of Doppler-based aortic evaluations and limited generalizability to other pediatric indications.

All included studies are summarized in Table 1.

**Table 1:** Summary of prospective studies (2018-2025) evaluating AI-assisted handheld or portable echocardiography performed by nonexpert operators A4C, apical four-chamber; AR, aortic regurgitation; AS, aortic stenosis; AUC, area under the curve; EF, ejection fraction; HF, heart failure; ICC, intraclass correlation coefficient; LV, left ventricle; MR, mitral regurgitation; PLAX, parasternal long-axis; RACE, Rapid Assessment of Competency in Echocardiography; RCT, randomized controlled trial; RV, right ventricle; TTE, transthoracic echocardiography

Study	Nonexpert users	Sample size	Device/AI feature	Key outcomes
Narang et al. (2021) [12]	Nurses (no prior echo training)	240 adults	Handheld ultrasound with real-time AI guidance (Caption Guidance)	Diagnostic adequacy: LV size/function and effusion 98.8%; RV size 92.5%
Mor-Avi et al. (2023) [13]	Nurses/residents	240 adults	Handheld scanner with real-time AI probe guidance	Diagnostic sufficiency: LV size 99.2%, LV function 99.6%, RV size 92.9%, effusion 100%; 83-96% concordance vs. experts; quantitative A4C feasible in 83%
Trost et al. (2025) [14]	Nurses (multicenter)	240 adults	Handheld system with AI probe guidance and auto-quality gating (HeartFocus)	10-view protocol completed in 100% of attempts; blinded review ≥95% diagnostic adequacy (LV size/function, RV size, effusion); auto-capture/quality gating yielded 99% diagnostically acceptable clips
Motazedian et al. (2023) [15]	Nonexperts vs. experts	449 enrolled (424 analyzed; 208 nonexperts, 216 experienced)	Handheld device with AI-automated EF analysis (FoCUS platform)	ICC 0.904 overall (nonexpert 0.921; expert 0.845); EF <50% AUC 0.98 (sensitivity 92.8%, specificity 92.3%); EF <30% AUC 0.99 (sensitivity 78.1%, specificity 98.0%)
Huang et al. (2024) [16]	Nurse-led HF clinic	100 adults	EchoNous Kosmos handheld with AI guidance, automated EF, and report generation (Us2.ai)	Interpretability 96%; EF <50%: AUC 0.88, sensitivity 84.6%, specificity 91.4%; mean absolute EF difference 6% vs. reference TTE; mean exam time 12.9 minutes
Gallant et al. (2025) [17]	ICU clinicians	10 patients (80 scans)	Handheld scanner (Exo Iris) with AI probe guidance and automated EF analysis	96% diagnostic PLAX/A4C within 5 minutes; median time 1.5 minutes (PLAX), 2.3 minutes (A4C)
Karni et al. (2025) [18]	Medical students	48 randomized	RCT; handheld device with real-time AI probe feedback vs. standard training	Apical view success 88% vs. 76%; modest increase in exam time (53 seconds); higher acquisition proficiency on standardized assessment
Baum et al. (2023) [19]	Internal medicine residents	43 randomized (22 AI, 21 control)	RCT; handheld device with AI guidance vs. standard training	Faster A4C acquisition (median 57 seconds vs. 85 seconds), higher blinded image quality (RACE score 4.5 vs. 2), and better recognition of reduced LV function (85% vs. 50%).
Peck et al. (2023) [20]	Nonexpert health workers (Uganda; pediatrics)	50 children	Handheld device with AI acquisition guidance and color Doppler	Diagnostic adequacy >90% for mitral valve morphology and MR; lower for aortic lesions (79% AR; 50% AS)

Discussion

What Works Well

Prospective studies across outpatient, acute, educational, and global settings consistently demonstrate that AI guidance enables nonexperts to acquire diagnostically adequate echocardiographic views after limited training. Multicenter adult trials report diagnostic adequacy exceeding 90% for LV systolic function and pericardial effusion, with strong agreement between nonexpert and expert references [12-14]. In high-acuity environments, ICU clinicians with minimal experience obtained diagnostic PLAX and A4C views in 96% of scans within minutes, supporting real-time decision-making in shock and respiratory failure [17]. In low-resource and pediatric contexts, health workers achieved reliable mitral-focused screening for RHD, illustrating feasible task-shifting where sonographers are scarce [20].

Automated EF further reduces dependence on operator experience. Multicenter evaluations have shown excellent correlation between AI-derived and reference EF values (ICC approximately 0.9; AUC 0.98 for EF < 50%), with comparable performance between nonexperts and experts once analyzable views were available [15]. In outpatient pathways, nurse-led AI protocols produced interpretable studies in nearly all patients within practical scan times [16]. These findings indicate that AI guidance and automation can elevate nonexpert performance into the diagnostic range and support efficient triage-oriented pathways.

What Remains Challenging

Despite substantial progress, several tasks remain technically difficult for both algorithms and operators. Apical outflow and other Doppler-dependent assessments, including aortic valve lesions, continue to show lower adequacy in nonexpert hands [20]. Poor acoustic windows arising from obesity, hyperinflation, or invasive ventilation further constrain image quality and therefore limit the reliability of automated analyses [15,17]. Some nonexpert clips remain unanalyzable despite guidance, which highlights that computation cannot substitute for fundamentally absent diagnostic information [15].

Overall, current evidence indicates that the main strengths of AI-assisted handheld echocardiography lie in its ability to enhance feasibility, consistency, and efficiency across nonexpert users, providing dependable visualization of core views and global function. However, Doppler-critical measurements remain the principal limitation, as both acquisition and alignment require nuanced operator input that is not yet fully replicable by algorithmic guidance. These tasks are therefore better reserved for expert or confirmatory imaging rather than frontline nonexpert workflows.

Implementation and Governance

Successful clinical adoption depends on structured onboarding, supervision, and transparent workflows. Nonexpert operators benefit from intuitive user interfaces with clear capture gates, quality indicators, and escalation prompts when AI outputs diverge from bedside findings. Programs should standardize documentation for limited POCUS studies, specify supervision and review thresholds, and maintain periodic blinded quality checks. These measures are aligned with existing handheld-POCUS governance statements, including recent interdisciplinary expert consensus recommendations [7].

Integration with institutional infrastructure is equally important. Interoperability with EHR and PACS systems, auditability of AI software versions, and accessible metadata on algorithm updates are essential for safety, accountability, and regulatory compliance [21].

Automation Bias and Mitigations

Automation bias, which refers to the undue acceptance of algorithmic outputs over clinical judgment, remains a recognized risk. Overreliance may occur when AI indicates a normal EF or absent effusion despite contrary clinical impressions. Practical safeguards include built-in confidence metrics, automatic supervisor alerts for discordant findings, and explicit prompts reminding users to correlate with clinical context. Training should emphasize that AI is a supportive rather than definitive decision tool. Future system designs should incorporate interpretability features, traceable output rationale, and user-feedback mechanisms to minimize cognitive complacency.

Strengths and Limitations of the Evidence Base

The collective evidence base demonstrates convergence of findings across diverse settings, including ambulatory, ICU, educational, and pediatric or low-resource environments [12-20]. Randomized trials strengthen confidence in the educational benefits of AI guidance [18,19], and several studies establish end-to-end nonexpert pathways with high interpretability and clinic-compatible examination times [16]. These convergent results across independent groups support the reproducibility and general feasibility of AI-assisted handheld echocardiography for core triage questions.

However, current evidence remains methodologically limited in several ways. Most studies focus on narrow diagnostic objectives such as LV systolic function and pericardial effusion, providing less insight into the broader range of echocardiographic assessments relevant to clinical practice. Many trials are small, single-center, or short in duration, which restricts statistical power and external validity. In addition, the widespread use of commercial AI platforms provided by manufacturers, with some degree of industry support or device provision, introduces potential bias. Most available data also assess intermediate outcomes such as image adequacy or correlation with expert interpretation rather than patient-centered or health-economic endpoints [12-20].

Strengths and Limitations of This Review

This review applied a structured PubMed search with predefined inclusion criteria focused on prospective studies involving nonexpert operators and AI-enabled handheld echocardiography. Citation chaining supplemented the search to ensure coverage of recent and pre-indexed trials.

The principal limitations include the narrative rather than meta-analytic synthesis, which reflects heterogeneity in study design, operator definitions, training intensity, device platforms, and adequacy thresholds. Retrospective and expert-only studies were excluded to maintain focus on nonexpert performance, although some may offer complementary insights outside this scope.

Future Directions

Future research priorities include pragmatic multicenter trials linking nonexpert AI POCUS to patient-level outcomes such as time-to-diagnosis, management changes, hospital admissions, length of stay, and mortality. Technical development should aim to expand validated nonexpert capabilities to include Doppler-aware guidance for outflow gradients, aortic valve disease, diastolic function, and congenital screening. Equity-focused research should ensure usability and performance across age, skin tone, and body habitus. Finally, governance frameworks integrating audit, algorithm version control, and longitudinal quality monitoring will be essential to ensure that AI-assisted echocardiography becomes a dependable, transparent, and equitable component of modern bedside practice.

## Conclusions

AI-assisted handheld echocardiography has moved from concept to practical reality for nonexpert operators. Across prospective studies, real-time guidance reliably elevates acquisition quality for core bedside questions, while automated EF analysis achieves expert-level agreement once analyzable views are available. These capabilities support faster triage in acute care, feasible nurse-led pathways in ambulatory settings, and task-shifted screening in pediatric and global contexts.

However, important boundaries remain. Performance is constrained by acoustic windows, and Doppler-dependent targets remain less robust in nonexpert hands. Moreover, patient-centered outcomes and cost-effectiveness have not yet been established at scale. With appropriate governance, targeted training, and interoperable integration into clinical systems, AI-enabled POCUS can broaden access to echocardiography, reduce diagnostic delays, and help address inequities in cardiovascular care. The central challenge now is deployment: implementing these tools safely, sustainably, and at scale so that essential cardiac imaging is available at the point of care for those who need it most.
